# Cerebral Venous Sinus (Sinovenous) Thrombosis in Children

**DOI:** 10.1016/j.nec.2010.03.006

**Published:** 2010-07

**Authors:** Nomazulu Dlamini, Lori Billinghurst, Fenella J. Kirkham

**Affiliations:** a The Hospital For Sick Children, 555 University Avenue, Toronto, ON M5G 1X8, Canada; b Neurosciences Unit, UCL Institute of Child Health, 30 Guilford Street, London WC1N 1EH, UK

**Keywords:** Cerebral sinovenous thrombosis, CSVT, Pediatric, Neonatal, Stroke

## Abstract

Cerebral venous sinus (sinovenous) thrombosis (CSVT) in childhood is a rare, but underrecognized, disorder, typically of multifactorial etiology, with neurologic sequelae apparent in up to 40% of survivors and mortality approaching 10%. There is an expanding spectrum of perinatal brain injury associated with neonatal CSVT. Although there is considerable overlap in risk factors for CSVT in neonates and older infants and children, specific differences exist between the groups. Clinical symptoms are frequently nonspecific, which may obscure the diagnosis and delay treatment. While morbidity and mortality are significant, CSVT recurs less commonly than arterial ischemic stroke in children. Appropriate management may reduce the risk of recurrence and improve outcome, however there are no randomized controlled trials to support the use of anticoagulation in children. Although commonly employed in many centers, this practice remains controversial, highlighting the continued need for high-quality studies. This article reviews the literature pertaining to pediatric venous sinus thrombosis.

Cerebral venous sinus (sinovenous) thrombosis (CSVT) is an increasingly recognized cause of childhood and neonatal stroke. Recent developments in the field highlight the expanding spectrum of perinatal brain injury associated with neonatal CSVT. Although there is considerable overlap in risk factors for neonatal and childhood CSVT, specific differences exist between the groups. Management remains controversial, unlike in adult sinovenous thrombosis. However, morbidity and mortality are significant, highlighting the continued need for high-quality studies within this field. This article reviews the literature on childhood CSVT (Table 1) and highlights developments in our understanding of neonatal CSVT.

## Epidemiology

More than 40% of childhood CSVT occurs within the neonatal period, with an incidence of 2.6 per 100,000 children per year in one series.^5^ The incidence of childhood CSVT varies between 0.4 and 0.7 per 100,000 children per year.^12,14^ These figures are probably underestimates of the true incidence for several reasons. Children with CSVT, particularly neonates, often present with nonfocal neurologic signs and symptoms, and the diagnosis may not be suspected.^12^ Old imaging techniques, the variable anatomy of sinovenous channels and rapid recanalization are all factors which may contribute to underdiagnosis. The lack of evidence supporting treatment and anxieties about safety of anticoagulation may also have reduced the impetus to make a diagnosis, particularly in suspected CSVT associated with hemorrhage.

## Anatomy and physiology of the venous system in neonates and children

The venous sinuses and veins lie within the subarachnoid space. Arachnoid villi project into the venous sinuses of the dura and are concentrated on the superior sagittal sinus, which is important for absorption and drainage of cerebrospinal fluid. Venous drainage is achieved by 2 systems: the superficial and the deep. The superficial drainage system is composed of the superficial cortical veins, superior sagittal sinus (SSS), torcula or confluence of veins, right transverse sinus (dominant in the majority of individuals), sigmoid sinus, and internal jugular vein. The deep venous system consists of the basal veins, which drain blood from the basal ganglia and germinal matrix in preterm neonates, the Galenic system with the 2 internal cerebral veins that form the vein of Galen, the straight sinus, the basal vein of Rosenthal, the torcula, and the typically nondominant left transverse sinus, which drains into the left sigmoid sinus and the left internal jugular vein.

The major venous outflow tracts include the internal jugular veins (IJV) and extrajugular collateral venous pathways such as the venous vertebral plexus and the extracranial emissary veins. In the supine position assumed by neonates, the IJV is the major venous outflow tract. However, in adult studies have shown that, in standing, the venous vertebral plexus is the main outflow tract. The extracranial emissary veins, are small, few, and not thought to play a major role in normal venous drainage. However, in certain conditions where there is congenital chronic venous outflow obstruction, such as craniosynostosis, they assume a central role providing an extracranial outflow pathway.^15,16^ In most infants, the cavernous sinus is not yet connected to the cerebral veins, resulting in less reserve and increased vulnerability within the venous drainage system.^15,17^

Positioning of the neonate has been shown to have a major influence on venous outflow. Neck flexion and compression of the SSS by the occipital bone have been implicated in the etiology of venous stasis and thrombosis,^18–20^ and is an area requiring further study.^21^

## Pathophysiological mechanisms

Thrombosis within the venous system results in outflow obstruction, venous congestion, and a consequent increase in capillary hydrostatic pressure, driving fluid into the interstitium and producing edema. A persistent increase in hydrostatic pressure may result in red blood cell diapedesis, and if in excess of arterial pressure, a reduction of arterial inflow and arterial ischemia can occur.

## Spectrum of brain injury in CSVT

**Fig. 1:**
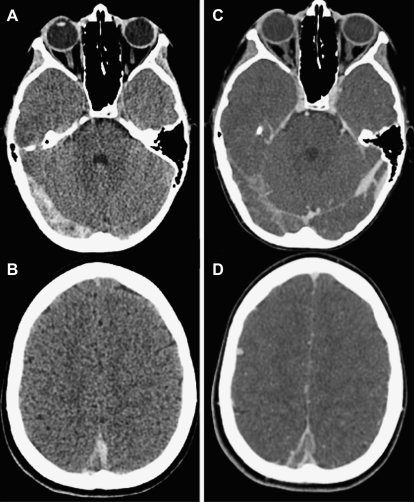

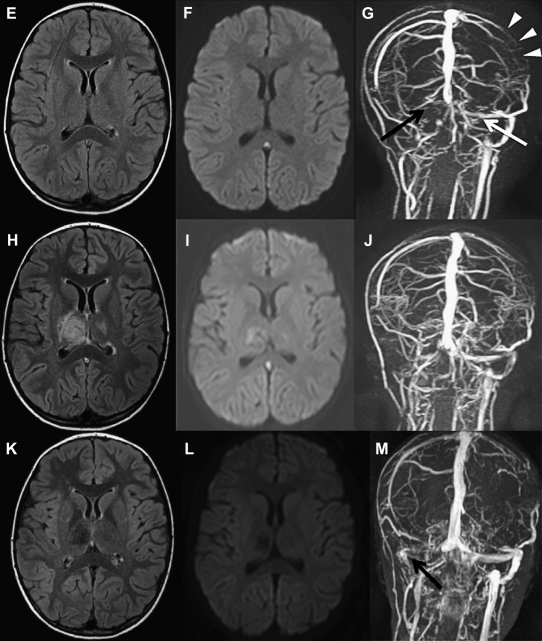
Case synopsis. A previously healthy 8-year-old girl was admitted with a 3-week history of, intermittent emesis and a 4-day history of occipital headache, and photophobia. Examination revealed severe dehydration, mild hypertension, and tachycardia. Extensive thrombosis of both deep and superficial cerebral sinovenous systems was diagnosed on head CT and anticoagulation therapy was initiated. Progressive encephalopathy developed on hospital day 5, necessitating admission to the intensive care unit. Unexplained tachycardia (heart rate >200) developed on hospital day 15 and Graves disease was ultimately diagnosed (thyrotropin <0.01 mIU/L and free T4 >77.2 pmol/L.) The patient was then started on methimazole. Comprehensive prothrombotic testing uncovered a heterozygous mutation in the Factor V Leiden gene. She completed 6 months of anticoagulation with subcutaneous low molecular weight heparin. Follow-up neurologic examination revealed mild left incoordination and bilateral kinetic tremor (left > right), perhaps secondary to hemorrhagic venous infarction of the right thalamus. (*A*, *B*) Non-contrast axial head CT done at admission revealed heterogeneous attenuation within the right transverse and sigmoid sinuses (*A*) and posterior aspect of the superior sagittal sinus (*B*), suggesting acute and subacute components of the thrombus. (*C*, *D*) Contrast CT reveals filling defects within these same sinuses. (*E*, *F*) Initial axial fluid-attenuated inversion recovery (FLAIR) (*E*), T1 and T2 (not shown) MRI sequences as well as diffusion-weighted imaging (DWI) (*F*) showed normal brain parenchyma. (*H*, *I*) A repeat MRI done in the subacute period after the patient's clinical deterioration showed increased signal within the thalami bilaterally on FLAIR (*H*) and T2 (not shown). Corresponding areas of diffusion restriction on DWI (*I*) suggested venous congestion and infarction secondary to thalamostriate venous occlusion. Peripheral blooming was seen in the right thalamus on gradient echo sequences (not shown), evidence of petechial hemorrhage. (*K*, *L*) Follow-up MRI done 6 months after diagnosis showed low FLAIR (*K*) and T2 signal (not shown) in the right thalamus, corresponding to hemosiderin deposits from hemorrhagic infarction. DWI (*L*) similarly showed low signal. (*G*, *J*, *M*) Three-dimensional phase contrast MR venograms performed acutely (*G*) and subacutely (*J*) showed extensive sinovenous thrombosis, involving the right transverse and sigmoid sinuses (*black arrow*), right internal jugular vein, posterior superior and inferior sagittal sinuses, torcula, vein of Galen, basal vein of Rosenthal, and internal cerebral and thalamostriate veins. Left parietal cortical veins were also thrombosed (*white arrowheads*). The left transverse and sigmoid sinuses were spared (*white arrow*). Interval recanalization of the left internal cerebral vein and basal vein of Rosenthal was seen subacutely (*J*). A 2-dimensional time-of-flight MR venogram done 6 months post diagnosis (*M*) showed persistently absent flow within the right transverse sinus, but partially visualized flow within the right sigmoid sinus and jugular bulb (*black arrow*), evidence of either partial recanalization or slow flow within these sinuses. There was complete recanalization of the superior sagittal sinus, deep venous system, and left parietal cortical veins.

**Fig. 2:**
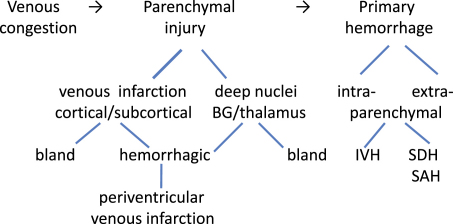
Spectrum of CSVT related brain injury. BG, basal ganglia; SDH, subdural hemorrhage; IVH, intraventricular hemorrhage; SAH, subarachnoid hemorrhage.

The spectrum of brain injury in CSVT varies from venous congestion, which may or may not be appreciable on neuroimaging (Fig. 1), to the more recognized parenchymal ischemic injury, which may be cortical or subcortical, and involve deep gray matter (see Fig. 1; Fig. 2). The majority of the parenchymal infarcts are hemorrhagic. Less well appreciated is CSVT-related primary subarachnoid and subdural hemorrhage. In preterm and term neonates there is also an association between intraventricular hemorrhage (IVH) and CSVT.^22^ Several studies demonstrate that CSVT is the most frequently recognized cause of symptomatic IVH, and is associated with basal ganglia and thalamic hemorrhage in term neonates. Deep venous thrombosis can be accompanied by hemorrhage into the ventricles as a result of blockage and hypertension in the deep venous drainage system.^10,23^ Presumed perinatal ischemic stroke is a subgroup of perinatal stroke and encompasses imaging-confirmed focal infarction, which may be venous or arterial, presenting after the neonatal period. Perinatal venous infarction (PVI) is one of these periventricular infarction syndromes, and is an underrecognized cause of congenital hemiplegia.^24^

## Risk factors for CSVT development

As is the case in adults, CSVT in neonates, infants, and children is often multifactorial in etiology, with a predisposing comorbid condition or infirmity identified in up to 95% of those affected (see Table 1). These conditions include common childhood illnesses such as fever, infection, dehydration, and anemia, as well as acute and chronic medical conditions such as congenital heart disease, nephrotic syndrome, systemic lupus erythematosus, and malignancy (Table 2). As well as the maternal, there are neonatal risk factors for sinovenous thrombosis in the perinatal period (Table 3), which parallel those in older children.

In addition to these systemic risk factors, thrombosis can develop and propagate in response to local venous stasis. A large number of children have coincident local head and/or neck pathology, including head trauma, central nervous system tumors, or recent intracranial surgery. Historically, CSVT was a well-recognized complication of otitis media and mastoiditis, and while less attention has been paid recently to this important risk factor, otitis media or mastoiditis has been identified in 24% to 62% of all childhood CSVT case series and cohorts published in the last decade.^1,5,7,13,27,28^ Indeed, in terms of observed frequency, infection appears to be the most common condition associated with CSVT in children outside of the neonatal period.

Anemia is frequently observed in children with CSVT, though mechanisms for its contribution to thrombus development are incompletely understood. Iron deficiency anemia and microcytosis are most commonly described^7,25,29–33^ sometimes in association with thrombocytosis, but only one study with parallel controls is currently available.^25^ CSVT has also been reported in chronic anemias, such as hemolytic anemia and Evans syndrome,^34^ β-thalassemia major,^35^ and sickle cell disease.^36–40^ The diagnosis of anemia may be obscured by relative hemoconcentration (particularly if dehydration is also present) and a falsely elevated ferritin in the acute setting, so it is important that the diagnosis of anemia and iron deficiency should be comprehensively excluded or treated in all children with CSVT.

Dehydration is another important treatable risk factor for pediatric CSVT, secondary either to increased fluid losses from nephrotic syndrome^30^ or gastroenteritis, or poor oral intake with infection or systemic medical illness. Dehydration and hypovolemia should always be carefully assessed and corrected to prevent thrombus propagation and promote recanalization of the affected vessel.

Other common illnesses, including meningitis^41^ and diabetes,^29^ may be complicated by CSVT, which can be difficult to diagnose so that data for incidence remain a minimum estimate.^28^ Although occasionally recognized, there are few data on the prevalence of CSVT in convulsive and nonconvulsive seizures and status epilepticus^42^ and otherwise unexplained hydrocephalus.^43^ CSVT may also be an important determinant of outcome in minor head injury,^44,45^ and in traumatic^11,46,47^ and nontraumatic coma (eg, secondary to cerebral malaria).^48^ Other infections more commonly seen in tropical countries (eg, neurocystercercosis), may also be associated with CSVT.^49^

Certain chronic conditions such as inflammatory bowel disease,^50^ systemic lupus erythematosus,^51^ Cushing syndrome,^52^ and thyrotoxicosis^53^ (see Fig. 1) appear to predispose to CSVT, which may present in unusual ways, including psychiatric manifestations.^54^

## Prothrombotic disorders that may be risk factors for CSVT in children

Prothrombotic states have been identified in 24% to 64% of children^5,7,28,55,56^ and in 20% of neonates^10^ with CSVT in recent series (see Table 1). However, these data are difficult to interpret as (1) the number and types of available prothrombotic tests have varied over the past 2 decades and vary between centers, (2) not all children have full prothrombotic profiles assessed, and (3) results may depend on the timing of testing. Indeed, acquired prothrombotic tendencies, such as protein C, protein S, and/or antithrombin deficiency secondary to infection or protein loss (eg, in nephrotic syndrome), may normalize on repeat investigation with resolution of the acute process. High factor VIII levels, which may be determined by genetic and acquired factors, are also common^7,57^ but there are currently no controlled data. Although there is evidence for an excess of genetic polymorphisms, the relative importance of the Factor V Leiden mutation is less clear in children than in adults.^5,56,58^ While uncommon, the prothrombin 20210 mutation does appear to be a risk factor for recurrence and should be excluded.^4^

Homocystinuria is a rarely described association,^59^ and homozygotes for the thermolabile variant of the methylene tetrahydrofolate reductase (MTHFR) gene may have an increased risk of CSVT.^60^ Hyperhomocysteinemia (which has been shown to be a risk factor in 2 case-controlled series in adults^61,62^) and its genetic determinants may be worth excluding or treating with folic acid and vitamin B_6_ and B_12_ supplementation, as this has few risks, but further studies will be important.

Apart from those with the prothrombin 20210 mutation, who should probably be anticoagulated in high-risk situations,^4^ there are few data on whether long-term treatment of any of the other prothrombotic disorders reduces the recurrence risk.^5,28^ Investigation for prothrombotic disorders is expensive and may not guide management except in certain circumstances, such as determining the risks of using oral contraception (see later discussion). Nevertheless, full prothrombotic profiles should be considered in all affected children, to better counsel parents of patients and also contribute data that may improve our understanding of mechanisms underlying CSVT development.

## Clinical presentation

The clinical manifestations of CSVT are nonspecific, and may be subtle in neonates and children (Table 4). Although rare, cerebral sinovenous thrombosis can occur antenatally as early as the second trimester and is detectable by fetal real-time and color Doppler ultrasound.^63^ Reported cases are likely an underestimation of frequency, as the imaging characteristics mimic those of an intracranial tumor. Thrombosis often occurs within the posterior fossa and may occur in association with dural malformations such as dural arteriovenous shunts. Spontaneous regression of the thrombosis may occur, with a favorable outcome. Diagnosis is important, as therapeutic terminations of pregnancy have resulted in misdiagnosis.^64^ The fetal venous drainage system may be less susceptible to thrombosis compared with the neonate, as fetal anastomoses may result in the fetus being able to redirect venous blood flow.^65^

Outside of the antenatal period most of the clinical scenarios occur at all ages, and the clinician requires a high index of suspicion to make the diagnosis. The clinical manifestations of CSVT are nonspecific, may be subtle (see Table 4), and may overlap with predisposing conditions such as infection and dehydration.^7,12^ Seizures, altered levels of consciousness and encephalopathy, focal neurologic deficits (cranial nerve palsies, hemiparesis, hemisensory loss), and diffuse neurologic symptoms (headache, nausea, emesis) may result. While most of the clinical symptoms can occur at any age, seizures are more common in neonates, and focal and diffuse neurologic signs are more common in older infants and children.^12^ The clinician should consider this diagnosis in a wide range of acute neurologic presentations in childhood, including those accompanied by neuroimaging evidence of hydrocephalus,^43^ subdural effusion or hematoma,^66^ subarachnoid hemorrhage,^67^ or intracerebral hemorrhage or infarction, particularly in the parietal or occipital regions.^7^ Presentation with pseudotumor cerebri^68^ and isolated headache^69^ have been well documented. A high index of suspicion is necessary to effect earlier detection and therapeutic strategies.

## Diagnosis

### Neuroimaging Techniques

The keys to neuroradiological diagnosis (Table 5) are (1) a high index of suspicion of the diagnosis in the acute phase so that imaging is performed early, as the venous sinuses may recanalize before detection,^4,7,70^ and (2) a good working relationship between treating clinicians and neuroradiologists so that definitive neuroimaging and investigations are pursued if necessary.

Anatomic and clinical studies demonstrate a link between venous drainage and location of parenchymal infarcts.^71,72^ Unenhanced computed tomography (CT) scans may detect deep venous thrombosis as linear densities in the expected locations of the deep and cortical veins (see Fig. 1A, B).^11,73^ As the thrombus becomes less dense, contrast may demonstrate the “empty delta” sign, a filling defect, in the posterior part of the sagittal sinus (see Fig. 1C, D).^11,28^ However, CT scan with contrast misses the diagnosis of CSVT in up to 40% of patients.^9,27,28^ Diffusion and perfusion magnetic resonance imaging (MRI) may play a role in detecting venous congestion in cerebral venous thrombosis (see Fig. 1H, I) and in the differentiation of cytotoxic and vasogenic edema, but does not differentiate venous from arterial infarction. CT venography or MRI with venography (MRV) are now the methods of choice for investigation of CSVT.^7,9,71,74^ The diagnosis is established by demonstrating a lack of flow in the cerebral veins (see Fig. 1G,J,M) with or without typical images of brain infarction (see Fig. 1E, F, H, I).

The superficial venous system is more frequently involved than the deep system, and the most common sites of CSVT are the transverse, superior sagittal, sigmoid, and straight sinuses. Between one- and two-thirds of children with CSVT may have parenchymal brain lesions such as venous infarction and hemorrhage.^71^ MRI and MRV are important in both the demonstration of the infarct and the clot within the vessels.^71^ On MRI, the thrombus is readily recognizable in the subacute phase, when it is of high signal on a T1-weighted scan, and MRV may not be required. In the acute phase, the thrombus is isodense with brain on T1-weighted imaging and of low signal on T2-weighted imaging. This appearance can be mistaken for flowing blood, but MRV will demonstrate an absence of flow in the thrombosed sinus. T2∗-weighted MRI seems to be more sensitive than T1- or T2-weighted or fluid-attenuated inversion recovery (FLAIR) imaging in demonstrating venous thrombosis and associated hemorrhage.^75,76^ However, MRI and MRV are techniques prone to flow artifacts (see Fig. 1M) and in equivocal cases, particularly if deep venous infarction or cortical venous thrombosis is suspected, an endoluminal technique such as high-resolution CT venography or conventional digital subtraction angiography may be required as a final arbiter.

## Investigation, monitoring, and management

Laboratory investigation of adult and pediatric CSVT is similar (Table 6). Treatment of CSVT (Table 7) has historically involved general supportive care or symptomatic measures, such as correction of dehydration and hypovolemia, antibiotics for cases involving infection, control of seizures with anticonvulsants, and medical and surgical measures aimed at decreasing intracranial pressure. In cases of otitis media-related and mastoiditis-related CSVT, many children receive parenteral antibiotic therapy, with either second- or third-generation cephalosporins (Table 8). Antibiotic choice and treatment duration in children with head and neck infections should be discussed with a local infectious disease specialist and consideration given to coverage with metronidazole, clindamycin, or vancomycin when anaerobic organisms are implicated (ie, *Fusobacterium necrophorum* in Lemierre syndrome or jugular venous thrombophlebitis).^42^ The role of surgery, such as mastoidectomy, myringotomy, and/or tympanostomy tube insertion, in otitis media-related and mastoiditis-related CSVT is unclear,^77^ but is often performed based on the preference of the treating otolaryngologist. Some patients develop intracranial hypertension within the clinical spectrum previously described as “pseudotumor cerebri” or “otitic” communicating hydrocephalus, and may require long-term acetazolamide therapy, serial lumbar punctures, or lumboperitoneal shunting (see section on Follow-up).

Pediatric case series published in the last decade differ in their reported use of antithrombotic agents after the diagnosis of CSVT is established. Treatment regimens vary between centers, but many older infants and children receive anticoagulation in the acute setting with either parenteral unfractionated heparin, subcutaneous low molecular weight heparin (LMWH), or oral warfarin (Coumadin; Bristol Myers-Squibb) (see Table 1). Some centers prefer to use unfractionated heparin acutely, as the effects of heparin can be reversed if intracranial hemorrhage occurs. This regimen is often followed by chronic anticoagulation with LMWH or Coumadin for 3 to 6 months. Anticoagulation should be carefully monitored, with activated partial thromboplastin time (APTT) for unfractionated heparin, anti-Xa for LMWH, or international normalized ratio for Coumadin, to achieve adequate levels for efficacy while preventing overdosage. However, anticoagulation may be terminated sooner than this if recanalization of the affected vessel(s) is demonstrated on follow-up neuroimaging with MR or CT venography. At some centers, there seems to be a reluctance to treat neonates with anticoagulation^12,13,78^ due to perceived risks of worsening preexisting intracranial hemorrhage or causing hemorrhagic transformation of bland venous infarction, coupled with a lack of evidence demonstrating improved outcome in neonates treated with anticoagulation. However, treatment of neonates with LMWH appears to be safe, and should at least be considered.^79^ Very few centers have reported on the use of antiplatelet agents such as acetylsalicylic acid (ASA)^7,12^ or dipyridamole in the acute or chronic^1^ settings.

There are currently no well-designed clinical trials in children to support acute or chronic antithrombotic therapy with anticoagulants or antiplatelet agents once the diagnosis of CSVT is made. The only randomized placebo-controlled trial of intravenous heparin in adults^80^ was stopped early because there was clear evidence of benefit, particularly in terms of mortality. Subsequent to this, a randomized placebo-controlled trial of subcutaneous LMWH in adults^81^ showed a trend for better outcome in the treated group, but the mortality was lower in this series and there were more patients with milder presentations in the placebo arm. Despite these limited data, a recent Cochrane review concluded that anticoagulation was safe, and there was some evidence for a clinically important benefit.^82^

Single-center and small multicenter series in children^7,57,74,83^ have shown that intravenous and subcutaneous LMWH can be used safely in children, with close monitoring of heparin levels or anti-Xa levels when LMWH is employed (see Table 8). DeVeber and colleagues^84^ initiated a prospective cohort study of anticoagulant therapy in 30 children with CSVT from 1992 to 1996, and reported a mortality rate of 3 out of 8 untreated compared with 0 of 22 treated children. One series suggested that cognitive outcome might be better in the anticoagulated group,^7^ and pooled data from the European collaborative group found a reduced risk of recurrence in those who were anticoagulated.^4^ In adult series, patients with hemorrhage were anticoagulated, and available evidence suggests that the benefit of anticoagulation on improved outcome outweighs the risk of new bleeding or extension of old hemorrhage. There is currently a consensus that in children beyond the neonatal period without hemorrhage, anticoagulation should be considered.^85–87^

There are no randomized data on thrombolysis,^1,3,88–90^ thrombectomy,^91^ or surgical decompression^92,93^ in CSVT even in adults,^94^ but each has been used with apparent success in isolated cases or small series of seriously ill patients, including children, usually in coma and with extensive thrombosis of superficial and deep venous structures.^79,88–90^ A nonrandomized study comparing urokinase thrombolysis with heparin in adults suggested better functional outcome for the thrombolysed patients but higher risk of hemorrhage.^95^ These patients have a high risk of secondary complications, including status epilepticus, hydrocephalus,^95^ and raised intracranial pressure,^96,97^ and may benefit from intensive care and monitoring of electroencephalograph and intracranial pressure as well as neuroimaging (see Table 8).

## Mortality and morbidity

CSVT-specific mortality is less than 10%, but neurologic deficits are present at time of discharge or follow-up examination in 17% to 79% of survivors, and motor and cognitive sequelae may require long-term rehabilitative regimens.^1,7,28,98–100^ Coma is a predictor of death in childhood CSVT.^7^ Most published pediatric cohorts have followed affected children for relatively short periods, typically less than 2 years from time of diagnosis. Despite aggressive therapy with antithrombotic agents, antibiotics, and surgery in some cases, many children with CSVT suffer chronic neurologic symptoms, such as headache, visual impairment, and cranial nerve VI palsy related to increased intracranial pressure. Others display deficits related to venous infarction ranging from developmental delays and learning disabilities to hemiparesis and hemisensory loss. In the series by Sébire and colleagues of children who presented at more than 1 month of age,^7^ older age, lack of parenchymal abnormality, anticoagulation, and lateral and/or sigmoid sinus involvement were independent predictors of good cognitive outcome, although the last predicted pseudotumor cerebri. More than 50% of neonates have a poor outcome, and mortality is high.^3,12^

## Follow-up

All children with CSVT require close monitoring for neurologic and ophthalmologic symptoms and signs related to increased intracranial pressure and optic nerve compression. As visual impairment and failure may go undetected by parents, particularly in nonverbal children, ophthalmology follow-up is warranted in the first year after diagnosis. Persistent headache, nausea, or vomiting (particularly if nocturnal or early morning) mandate repeat neuroimaging to exclude hydrocephalus, CSVT propagation, and/or recurrence. Chronically elevated intracranial pressure may respond to treatment with steroids or acetazolamide, or may require lumboperitoneal shunting.^94,101,102^ Occasionally patients with cryptogenic CSVT later manifest symptoms of an underlying disease (see Fig. 1), such as systemic lupus erythematosus or Behçet disease,^103^ so patients should be encouraged to report back if they have any other medical concerns after diagnosis.

Follow-up neuroimaging with MR or CT venography should be undertaken in the acute phase and during the first year of follow-up to look for evidence of extension or persistence or recanalization of venous occlusion, or the development of venous stenosis. Some centers perform this at 3, 6, and 12 months after diagnosis. In the European study, complete and partial recanalization occurred in 46% and 42%, respectively.^4^

## Prediction and prevention of recurrence

Between 10% and 20% of children who have a cerebral venous sinus thrombosis will experience a recurrent symptomatic venous event, at least half of which are systemic rather than cerebral (Table 9).^4,5,7,28^ In a multicenter European study,^4^ recurrent venous thrombosis only occurred in children whose first CSVT was diagnosed after age 2 years; the underlying medical condition had no effect. In Cox regression analyses, nonadministration of anticoagulant before relapse (hazard ratio [HR] 11.2, 95% confidence interval [CI] 3.4–37.0; *P*<.0001), persistent occlusion on repeat venous imaging (HR 4.1, 95% CI 1.1–14.8; *P* = .032), and heterozygosity for the G20210A mutation in factor II (HR 4.3, 95% CI 1.1–16.2; *P* = .034) were independently associated with recurrence. Among patients who had recurrent CSVT, 70% (15) occurred within 6 months after the initial episode.

There have been no trials of strategies to prevent recurrent cerebral or systemic venous thrombosis in children, but these cohort data suggest that anticoagulation should be considered for up to 6 months after the first episode. It would be difficult to recommend a higher risk strategy, such as prolonged oral anticoagulation, unless recurrence had already occurred, but there is a case for anticoagulation in acute settings where the risk of recurrence is likely to be high, for example, relapse of nephrotic syndrome or active inflammatory bowel disease.^4^ There is also a little evidence that stopping the use of oral contraceptives reduces the risk, and there are several low-risk strategies, such as improving the quality of the diet, which can be recommended (see Table 9).

## Summary

Cerebral sinovenous thrombosis is an underdiagnosed but important cause of stroke in childhood occurring most often in the neonatal period. Mortality and morbidity are significant. However, there are several unanswered questions regarding CSVT, particularly in relation to diagnosis in children presenting with hydrocephalus, or in coma or status epilepticus in the context of common conditions such as head injury, as well as the safety and efficacy of treatment in this age group. Hence the need for further high quality studies and where possible - well conducted randomized controlled trials.

## Figures and Tables

**Table 1:** Pediatric CSVT literature summary

Study	No. of patients	Demographics, N (%)			Risk Factors				Infarction (%)	Treatment (%)		Outcome (%)		
		Country	Males	Neonate	None, N (%)	Systemic (N or %)	Infection (%)	PT (%)		Acute ACT	Chronic ACT	Follow-up (y)	Death	Abnormal
Mallick et al, 2009^1^	21	UK	10 (48)	0	2 (10)	Nephrotic syndrome (3)	Any infection (71)	25	14	100	67	0.42–6	10	29
						CNS tumor (1)	OM/Mastoiditis (62)		Bland (100)	UFH (100)	Coumadin (100)			
						OCP (2)	Sepsis (10)		Hemorrhagic (0)	LMWH (14)	LMWH (19)			
						Dehydration (14)								
						Anemia (19)								
Vieira et al, 2009^2^	53	Portugal	30 (57)	6 (11)	7 (13)	Nephrotic syndrome (2)	Any infection (57)	40	NR	68	100	1.1–6	0	43
						CNS tumor (1)	Mastoiditis (43)				Coumadin (100)			
						SLE (1)	Meningitis (13)							
						Head trauma (1)								
						Diabetes (1)								
						Chemotherapy (5)								
						Dehydration (4)								
Wasay et al, 2008^3^	70	USA	28 (40)	25 (36)	7 (10)	Nephrotic syndrome (1)	Any infection (40)	56	NR	21	12	NR	13	46
						SLE (2)	OM/MA/Sinusitis (24)				Coumadin (100)			
						SCD (1)	Meningitis (3)							
						Homocystinuria (3)	Sepsis (13)							
						Leukemia (2)								
						OCP (1)								
						Chemotherapy (1)								
						Dehydration (4)								
						Anemia (10)								
						Fever (33)								
Kenet et al, 2007^4^	396	Germany	236 (60)	75 (19)	NR	NR	NR	NR	10	63	42	0–7.1	3	NR
		Israel							Bland (10)	UFH (51)	LMWH (76)			
		UK							Hemorrhagic (90)	LMWH (48)				
		Belgium												
Fitzgerald et al, 2006^5^	42	USA	24 (57)	42 (100)	NR	Cardiac condition (11)	Any infection (17)	64	60	7	0	0.2–15	3	79
						Dehydration (26)	Meningitis (10)		Bland (12)					
							Sepsis (7)		Hemorrhagic (88)					
									IVH (20)					
Bonduel et al, 2006^6^	38	Argentina	27 (71)	NR	3 (8)	SLE (1)	Any Infection (50)	NR	NR	68	68	0.25–11.5	23	32
						CNS tumor (2)				LMWH (68)	Coumadin (100)			
						Leukemia (8)								
						Lymphoma (2)								
						Head trauma (2)								
						Chemotherapy (7)								
						Dehydration (5)								
Sébire et al, 2005^7^	42	UK	27 (64)	NR	0	Cardiac condition (2)	Any infection (55)	62	60	43	43	0.5–10	12	62
						IBD (1)	OM (41)		Bland (52)	UFH (83)				
						Nephrotic syndrome (3)	MA (26)		Hemorrhagic (48)	LMWH (17)				
						SLE (2)								
						SCD (2)								
						Thalassemia (1)								
						CNS tumor (2)								
						Leukemia (2)								
						Dehydration (19)								
						Anemia (19)								
Kenet et al, 2004^8^	46	Israel	29 (63)	8 (17)	7 (15)	Cardiac condition (4)	Any infection (39)	42	NR	88	NR	NR	4	17
						IBD (1)	MA/Sinusitis (35)							
						SLE (2)								
						Homocystinuria (1)								
						OCP (1)								
						Head trauma (4)								
Barnes et al, 2004^9^	16	Australia	8 (50)	0	NR	NR	Any infection (88)	31	NR	63	NR	0.02–5	NR	38
							OM/MA (44)			UFH (30)				
							Meningitis/Abscess (44)			LMWH (80)				
										Coumadin (30)				
Heller et al, 2003^5^	149	Germany	84 (56)	40 (27)	44 (30)	IBD (1)	Any infection (44)	56	NR	88	73	NR	0	NR
						Nephrotic syndrome (1)	OM (3)			UFH (47)	LMWH (100)			
						Steroid use (3)	MA (9)			LMWH (40)				
						OCP (4)	Meningitis (4)							
						Head trauma (10)	Sepsis (5)							
							Sinusitis (3)							
							Varicella (1)							
							Gastroenteritis (3)							
Wu et al, 2002^10^	30	USA	NR	30 (100)	4 (13)	Cardiac condition (7)	Any infection (13)	57	NR	NR	NR	NR	NR	NR
						Dehydration (3)	Sepsis (10)							
							Pneumonia (3)							
Huisman et al, 2001^11^	19	Switzerland	9 (47)	0	NR	Head trauma (9)	Any infection (37)	NR	11	NR	NR	NR	11	NR
							MA (32)							
							Meningitis (5)							
DeVeber et al, 2001^12^	160	Canada	87 (57)	69 (43)	4 (3)	Cardiac condition (8)	Any infection (27)	24	41	53	NR	0.05–5.2	8	38
						Dehydration (25)	Sepsis (18)		Bland (32)	LWMH (59)				
									Hemorrhagic (68)	UFH (41)				
										Coumadin (46)				
Carvalho et al, 2000^13^	31	USA	21 (68)	19 (61)	NR	Cardiac condition (4)	Any infection (39)	NR	48	0	0	NR	13	52
						CNS tumor (1)	MA (23)							
						Chemotherapy (1)	Meningitis (10)							
						Dehydration (13)	Sepsis (7)							

All studies with more than 10 patients published since 2000 are included. *Abbreviations:* ACT, anticoagulation; APTT, activated partial thromboplastin time; CNS, central nervous system; IBD, inflammatory bowel disease; IVH, intraventricular hemorrhage; LMWH, low molecular weight heparin; MA, mastoiditis; NR, not reported; OCP, oral contraceptive use; OM, otitis media; PT, prothrombotic tendency; SCD, sickle cell disease; SLE, systemic lupus erythematosus; UFH, unfractionated heparin.

**Table 2:** Conditions associated with pediatric cerebral sinovenous thrombosis

General
Dehydration
Infection
Fever
Hypoxic-ischemic injury
Post lumbar puncture
Head and neck infections
Otitis media and mastoiditis
Meningitis
Sinusitis
Upper respiratory tract infection
Other head and neck disorders
Head injury
Post intracranial surgery
Hydrocephalus (±ventriculoperitoneal shunt)
Anemia
Iron deficiency
Sickle cell disease
Thalassemia
Autoimmune hemolytic anemia
Paroxysmal nocturnal hemoglobinuria
Autoimmune disorders
Behçet disease
Systemic lupus erythematosus
Antiphospholipid antibody syndrome
Inflammatory bowel disease (ulcerative colitis, Crohn disease)
Thyrotoxicosis
Cushing syndrome
Idiopathic thrombocytopenic purpura
Malignancy
Leukemia
Lymphoma
Central nervous system tumors
Cardiac disease
Cyanotic congenital heart disease^25,26^
Post-operative
Postcatheterization
Renal disease
Nephrotic syndrome
Hemolytic-uremic syndrome
Drugs
l-Asparaginase
Oral contraceptives
Corticosteroids
Epoetin-α
Chromosomal disorders
Down syndrome
Metabolic conditions
Diabetic ketoacidosis
Homocystinuria

**Table 3:** Conditions associated with neonatal cerebral sinovenous thrombosis

Maternal conditions
Chorioamnionitis
Diabetes
Hypertension
Perinatal conditions
Meconium aspiration
Apgar <7 at 5 min
Intubated at birth
Neonatal infection
Polycythemia
Severe dehydration
Pneumonia
ECMO treatment
Congenital heart disease
Disseminated intravascular coagulation
Congenital diaphragmatic hernia

*Abbreviation:* ECMO, extracorporeal membrane oxidation.

**Table 4:** Symptoms and signs of cerebral sinovenous thrombosis in older children

Seizures (focal, generalized)
Depressed level of consciousness and coma
Lethargy
Nausea
Vomiting
Headache
Visual impairment (transient obscurations, reduced acuity, blindness)
Papilledema
Hemiparesis
Hemisensory loss
Ataxia
Speech impairment, mutism
Cranial nerve palsies (VI)
Acute psychiatric symptoms
Respiratory failure (in neonates)
Jittery movements (in neonates)

**Table 5:** Diagnosis of sinovenous thrombosis

	Level of Evidence
High index of suspicion in children with associated pre-existing disorder	IC
High index of suspicion in children presenting with headache, seizures, coma	IC
Plain CT	IC
MRI (T1-, T2-weighted, T2∗, FLAIR)	IC
MRI with contrast	IIC
Diffusion-weighted MRI	IIC
CT venography	IIC
MR venography	IIC
Contrast MR venography	IIC
Transcranial Doppler	IIC
Conventional digital subtraction angiography	IIC

*Abbreviations:* CT, computed tomography; FLAIR, fluid-attenuated inversion recovery; MR, magnetic resonance; MRI, magnetic resonance imaging.

**Table 6:** Laboratory investigations in cryptogenic cerebral venous sinus thrombosis

	Level of Evidence
Essential	
Blood culture	IC
Full blood count	IC
Iron studies	IC
Thyroid function	IC
Antinuclear antibody or DNA binding	IC
Potentially useful	
Homocysteine	IIB
Vitamin status, ie, folate, B_6_, B_12_	IIB
Full prothrombotic screen (DNA and citrated samples)	IIB

**Table 7:** Acute management

	Level of Evidence
Supportive treatment	
Rehydration	IC
Treat infection, eg, antibiotics for meningitis/mastoiditis/pharyngitis	IC
Treat cause, eg, mastoidectomy, steroids for SLE, inflammatory bowel	IC
Treat seizures	IC
Treat iron deficiency	IIB
Anticoagulate/monitor for 4 months whether or not there is hemorrhage	
IV heparin/APTT	IIB
SC heparin/Factor Xa	IIC
Warfarin/INR	IIC
Thrombolysis	IIC
Thrombectomy	IIC
Surgical decompression	IIC

*Abbreviation:* INR, international normalized ratio.

**Table 8:** Monitoring of child with acute sinovenous thrombosis

	Level of Evidence
Clinical seizures (duration, semiology)	IC
Level of consciousness (Glasgow Coma Scale adapted for children)	IC
Focal neurologic signs, eg, hemiparesis	IC
Visual acuity and fields	IC
For those on intravenous heparin, 4-hourly APTT	IC
For those on subcutaneous heparin, daily factor Xa	IC
For those who are unconscious and/or ventilated:	
Continuous EEG monitoring	IIC
Intracranial pressure monitoring	IIC
Repeat neuroimaging	IIC

**Table 9:** Management of risk factors to prevent recurrence

	Level of Evidence
Improve diet, eg, 5 portions of fruit and/or vegetables per day	IC
Reduce cow's milk intake and increase solids in infants and toddlers	IC
Treat cause, eg, steroids for SLE, IBD	IC
Suggest alternative contraception	IB
Treat iron deficiency	IIC
Treat hyperhomocysteinemia/frank vitamin deficiency, eg, folate, B_6_, or B_12_	IIC
Consider acute anticoagulation in high-risk settings	IIA
Consider prolonged oral anticoagulation after recurrence	IIC
